# Permanent Maxillary Lateral Incisors’ Agenesis Managed by Mini-Screw Implant-Supported Pontics: A Scoping Review

**DOI:** 10.3390/dj13030096

**Published:** 2025-02-24

**Authors:** Elena Caramaschi, Elisabetta Lalli, Valentino Garau, Alessio Verdecchia, Enrico Spinas

**Affiliations:** 1 Department of Surgical Sciences, Postgraduate School in Orthodontics, University of Cagliari, 09124 Cagliari, Italy; caramaschi.elena@gmail.com (E.C.); dottoressa.lalli@gmail.com (E.L.); 2 Department of Surgical Sciences, Postgraduate School in Oral Surgery, University of Cagliari, 09124 Cagliari, Italy; valentino.garau@unica.it

**Keywords:** agenesis, maxillary lateral incisors, mini-screw, transitional restoration

## Abstract

**Background/Objectives:** The Agenesis of maxillary lateral incisors occurs with a variable prevalence in different ethnic groups, and there is a need for a temporary replacement until maturity has been reached in patients for whom the replacement solution has been chosen. This study aims to analyze the scientific evidence available to date concerning the use of mini-screw implant (MSI)-supported pontics for the transitional management of permanent maxillary lateral incisors’ agenesis in developmental age subjects. **Methods:** Electronic research was conducted using four databases: PubMed, Clarivate Analytics/Web of Science Core Collection, Scopus, and the Wiley/Cochrane Library. Six studies were included in the final review. Data were extracted based on the first and second author, year of publication, study design, sample characteristics, mini-screw implant (MSI) characteristics, MSI insertion and loading protocol, characteristics of the prosthetic component, and outcomes during the follow-up time. **Results:** Clinical outcomes were proven positive in all studies. In only one study did MSIs show mobility and consequent failure after one month. Discoloration of the prosthetic part proved to be the main complication. **Conclusions:** The comparison with conventional removable prostheses and fixed dental prostheses revealed that MSI-supported pontics are a viable alternative and a promising temporary solution until the end of growth. Further studies to standardize protocols and assess long-term outcomes are needed.

## 1. Introduction

One of the most common developmental anomalies in humans is the hypodontia of one or more permanent teeth [1]. The most affected elements are the third molars, followed by the mandibular second premolars, maxillary lateral incisors, and maxillary second premolars [2]. Maxillary lateral incisors are among the most frequently missing teeth [3,4,5], with a prevalence that generally ranges between 1.91% and 3.6% [6,7].

In adolescent patients, agenesis of maxillary lateral incisors leads to aesthetic and functional issues such as diastemas, midline discrepancy, and mesial migration of maxillary canines with consequent loss of canine guidance. The self-esteem of these young patients can also be affected [7].

In the case of congenital unilateral or bilateral absence of maxillary lateral incisors, there are various treatment possibilities. Both advantages and disadvantages are present in the therapeutic choice of opening spaces and closing spaces. Important factors to consider in choosing the most appropriate treatment strategy include the patient’s age, the type of malocclusion, the relationship of the anterior teeth, facial profile, the size, shape, and color of canines, as well as the height of the smile line [8]. Each treatment option, such as canine substitution for lateral incisor, prosthetic restorations like fixed and removable partial dentures, dental implants, and tooth autotransplantation, comes with its own limitations [9,10,11,12].

In children and adolescents, in the case of choosing to open spaces and maintain the space until growth is completed, it is necessary to resort to provisional restorations after orthodontic treatment and until growth is fully achieved [13]. Prosthetic rehabilitation in growing patients is routinely performed in clinical practice and as soon as possible to minimize aesthetic and phonetic disturbances [14]. The choice to use fixed prosthetics often requires the reduction of enamel from adjacent healthy teeth and is frequently associated with complications such as debonding [15]; therefore, the treatment of choice for growing patients refers to removable prosthetic devices. The exposure of the edentulous space upon removal of these appliances can create aesthetic problems in young patients [10,16]. Moreover, the literature indicates an increase in the incidence of caries, periodontal complications, and atrophy of the residual alveolar bone due to the lack of loading [17,18,19].

Osteointegrated dental implants are contraindicated in growing patients due to the risk of implant submersion after the eruption of surrounding teeth [11,20]. Mankani et al. recommend placing a dental implant after the completion of craniofacial and dental growth [19].

As an alternative procedure for the temporary replacement of missing maxillary lateral incisors in growing patients, mini-screw implants (MSIs) have been proposed [13], commonly used for orthodontic anchorage. MSIs have lower roughness and surface area compared to standard implants, significantly reducing the chances of osseointegration [11,21,22]. In addition to this significant concept, there are other potential advantages, such as the stimulation of bone remodeling, preservation of alveolar bone volume, and the maintenance of the root space created through orthodontic treatment [16,23]. The cost of MSIs is lower compared to standard implants, and the placement procedures are simplified due to the flapless surgical technique [24]. MSI-supported pontics enhance masticatory efficiency, speech, and patient comfort when compared to conventional prostheses [25]. Once growth is complete, the removal of the MSIs is straightforward, and a conventional implant can be inserted immediately after its removal [26]. In literature, it has been demonstrated that MSIs exhibit varying amounts of bone-to-screw contact [27], and this, in growing patients, could lead to infraocclusion of the MSIs [28]. To date, the use of MSIs during craniofacial growth appears to be a controversial topic [19].

Considering these conditions, the aim of this study is to investigate the current available evidence (clinical studies and case reports) and analyze the short-, medium-, and long-term survival of MSI-supported pontics for the transient management of agenesis of maxillary lateral incisors.

## 2. Materials and Methods

### 2.1. Protocol and Registration

This review was conducted in adherence to the protocol established by the Preferred Reporting Items for Systematic Reviews and Meta-Analyses extension for conducting Scoping Reviews (PRISMA-ScR) [29]. This scoping review was not registered.

To define the parameters of the research strategy, we formulated the following target question: “Is there a potential contribution of MSI intervention in managing missing permanent lateral incisors in growing patients compared to conventional removable prosthesis and fixed dental prosthesis treatment?”.

The studies were included if they met the criteria reported in the P.I.C.O. format:

P.: studies involving growing human subjects presenting with at least one agenesis of permanent maxillary lateral incisors;

I.: studies concerning the use of mini-screw implant (MSI)-supported pontics for the transitional management of permanent maxillary lateral incisors’ agenesis;

C.: studies comparing conventional removable prosthesis and fixed dental prosthesis with MSI-supported pontics;

O.: studies assessing alveolar preservation.

### 2.2. Information Sources and Search Strategy

A comprehensive systematic search was conducted in the following databases: PubMed, Clarivate Analytics/Web of Science Core Collection, Scopus, and the Wiley/Cochrane Library. Additionally, a grey literature search was performed using OpenGrey to ensure broader coverage. The search timeframe spanned from the inception of each database to December 2024. The search strategy included a combination of keywords and free-text terms related to (synonyms of) “orthodontic mini-implants”, “missing lateral incisor”, and “transitional restoration”. A detailed overview of the search strategies developed for each database is presented in Table 1.

### 2.3. Study Selection and Eligibility Criteria

An independent electronic search was conducted by two independent examiners [E.C. and E.L.]. In the case of disagreement, a third reviewer [A.V.] resolved the issue. No language limit was applied in the search strategy. No time limit was applied to the search. The inclusion criteria for the selection of articles were as follows: (1) growing human patients with at least one congenitally missing permanent maxillary lateral incisor, (2) use of mini-screw implant (MSI)-supported pontics, (3) studies with follow-up after the placement of the transitional restoration, (4) studies with radiographic and clinical assessment (5) clinical trials, (6) case report/case series, (7) randomized controlled trials, and (8) retrospective and prospective studies. The exclusion criteria were as follows: (1) patients with full deciduous dentition, (2) studies regarding adult patients, (3) studies in subjects with systemic/genetic disease, and (4) letters to the editor, commentaries, and reviews.

### 2.4. Data Extraction and Synthesis

Two different reviewers (E.C. and E.L.) elaborated the data-charting form. The same two reviewers extracted the data independently. The following characteristics have been selected in the data extraction: first author, year of publication, study design (type of the study), patients’ characteristics (gender and age), agenesis’ number and site, prosthetic-related characteristics MSI-supported pontics, loading timing, retention protocol, follow-up period, outcomes and complications. The extracted outcomes were retrieved from the included studies and then summarized. The inter-rater reliability analysis revealed a level of agreement between the authors of 0.68 of Cohen’s Kappa coefficient [30].

## 3. Results

### 3.1. Literature Search and Screening Process

**Figure 1:**
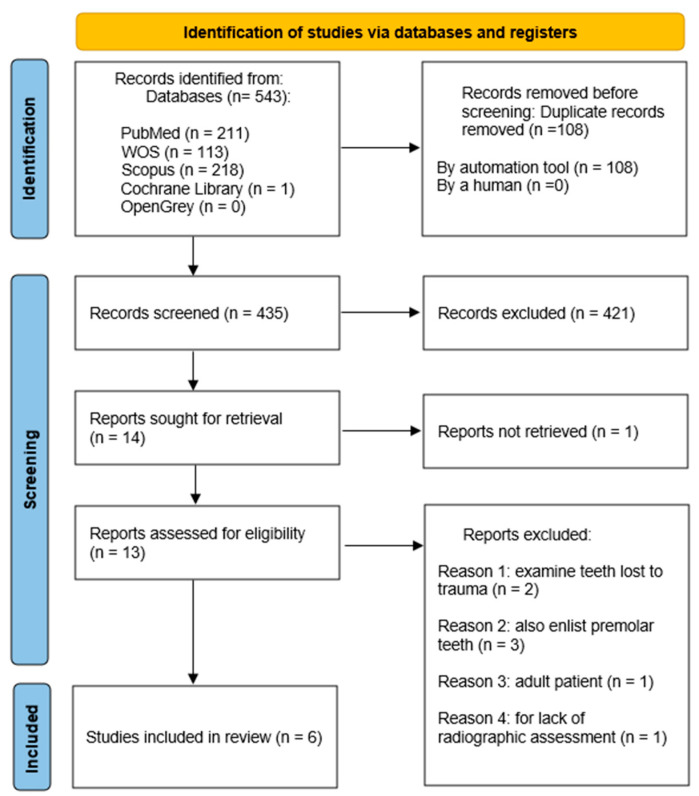
Flow chart of the search strategy and selection of articles to be included.

A total of 543 publications were identified from the PubMed (*n* = 211), Web of Science (*n* = 113), Scopus (*n* = 218), and Cochrane Library (*n* = 1) databases. No studies were identified in the grey literature search using OpenGrey (*n* = 0). A total of 108 studies were eliminated due to duplicate removal. Following the removal of duplicates, 435 records remained. The titles and abstracts of 435 articles were analyzed, and an additional 421 articles were excluded. Subsequently, the full text of 13 out of the remaining 14 studies was reviewed; 1 study could not be retrieved. A manual search in the references lists of the 13 selected articles resulted in no one additional article. After a full-text review, seven articles were further not included. Two studies were excluded due to examining teeth lost to trauma, three for enrolling patients with missing premolar elements in the examination, one because they treated an adult patient, and one for lack of radiographic assessment. In the end, six studies were included that met the inclusion criteria for this scoping review. A summary of the literature search and selection procedures was depicted in a flowchart in Figure 1.

### 3.2. Description of the Included Studies

Table 2 describes the main characteristics of the studies included in this scoping review. The studies were published between 2007 [23] and 2023 [31,32]. Two studies were recent, being published in 2023 [31,32]; three were published in 2014, 2015, and 2017 [11,13,16], respectively; only one study was published in 2007 [23].

#### 3.2.1. Study Design

Three studies were case series [11,13,23], and three were case reports [16,31,32], according to the study design.

#### 3.2.2. Sample Characteristics

None of the selected studies (*n* = 6) included a control group for comparison [11,13,16,23,31,32]. The patients’ age ranged from 10 to 16 years. The sex of patients was both female and male, with a higher prevalence of female patients. Two patients presented bilateral agenesis of the upper lateral incisors [13,32], while nine had unilaterally missing permanent maxillary lateral incisors [11,13,16,23,31].

#### 3.2.3. Mini-Screw Implant and Prosthetic Component Characteristics

Table 3 describes the mini-screw implant and prosthetic component characteristics of the selected studies.

##### Type of MSI

The diameter of the MSIs was variable in the included studies. This ranged from a minimum of 1.4 mm [16] to a maximum of 2.5 mm [31]. Only one study [13] did not report the MSIs’ diameter.

In contrast, the length of MSIs was less varied. It was 8 to 10 mm in four studies [16,23,31,32], and the remaining two studies did not specify it [11,13].

##### Type of MSI Positioning and Insertion Protocol

The MSIs were inserted perpendicular to the alveolar ridge in all studies, except for Ciarlantini and Melsen [13], who oriented the MSIs horizontally and palatally. The MSI insertion protocol was described in four of the six studies reviewed [11,23,31,32]. A full-thickness flap elevation under local anesthesia was performed by Rathi et al. [31] and Cope and McFadden [11], whereas in two studies [23,32], a flapless technique was used.

##### Type of MSI Loading

Ciarlantini and Melsen, Graham and Cope, and McFadden [11,13,23] loaded the MSIs immediately. Only Saha et al. [32] opted for mini-screw loading after 4 weeks. Two studies [16,31] did not report the loading protocol.

##### Type of Prosthetic Retention

Temporary cement was used for prosthetic retention in two studies [11,31], and in only one study, permanent cement was used [16]. Ciarlantini and Melsen and Graham opted for resin bonded [13,23]. The type of cement was not specified in Saha’s study [32].

##### Type of Prosthetic Component

The material of choice for the prosthetic component was composite resin [13,31,32]. Two studies [11,16] opted for ceramic crowns. Cope and McFadden [11], in the first clinical case, first rehabilitated with a resin crown and then replaced it with a ceramic crown; in the other patient, they immediately opted for ceramic prosthetic rehabilitation. Kalia [16] also chose an immediate ceramic crown.

##### Type of Retention

Regarding the type of retention, clear plastic retainers and lingual fixed retainers (including the pontic) were used in one study [23]. In one study, a maxillary fixed retainer was performed that did not include the MSI crown [16]. Ciarlantini and Melsen splinted the canine and the central incisor with the adjacent teeth but not with the pontic [13]. Saha used a Hawley restraint plate [32]. Two studies did not say anything about the use and type of retention [11,31].

### 3.3. Outcomes

Table 4 describes the outcomes of the case series/reports included in the study.

#### 3.3.1. Follow-Up Time

Assessing the follow-up of the studies under consideration revealed a significant variability. Cope and Mc Fadden reported the longest follow-up period: 8 years [11], while Graham’s study is the one with the shortest follow-up [23].

#### 3.3.2. Clinical Outcome

The clinical outcomes showed the presence of MSIs’ stability [11,13,16,23,31,32]. Only Graham reported the mobility of a mini-screw one month after insertion and subsequent failure [23]. Two studies showed no soft tissue inflammation [31,32]. In four studies, no marginal bone loss was detected [11,13,16,31]. Cope and Mc Fadden and Ciarlantini and Melsen observed the coronal bone growth without infraocclusion [11,13]. A favorable aesthetic outcome was also reported by Kalia [16].

#### 3.3.3. Complications

The most encountered complication concerns prosthetic rehabilitation, which becomes discolored over time [11,13]. Ciarlantini and Melsen [13] reported gingival impingement in two patients, and one study reported the formation of 1 mm diastema between the upper central incisors [32]. No notable complications were mentioned in three studies [16,23,31].

## 4. Discussion

This scoping review offers a comprehensive analysis of the available evidence regarding the use of mini-screw implant (MSI)-supported pontics for managing permanent maxillary lateral incisor agenesis in growing patients.

The included studies uniformly demonstrated favorable clinical outcomes associated with MSI-supported pontics. The stability of the implants, minimal marginal bone loss, and the absence of infraocclusion were among the most frequently reported findings [11,13,16,23,31,32]. These results align with existing evidence supporting the capacity of MSIs to maintain alveolar bone integrity and stimulate bone remodeling during craniofacial growth. Such benefits are particularly critical in growing patients, where bone preservation is essential for subsequent definitive restorations [13,23].

The alveolar process develops and regresses following the eruption and loss of teeth. The preservation of the bone depends on the presence of teeth. The topography of the alveolar bone includes the cortical plate and central portion of spongy bone. The alveolar bone undergoes continuous remodeling thanks to the osteoblast activity during growth, osteocytes that act as mechanosensors to detect forces from chewing and transmit signals to coordinate remodeling, and osteoclasts that resorb bone and the fibroblast that produce and maintain the connective tissue and secrete growth factors [33]. In patients with maxillary lateral incisors agenesis, the dimension of the alveolar ridge is already presented as reduced if compared to control groups, so it is fundamental to preserve the bone integrity as much as possible [34]. The MSI-supported pontics can give mechanical loading that stimulates remodeling and bone maintenance. There are various hypotheses supported by meta-analyses about the effects of increasing load on the maintenance of bone mineral density [35]. Wyatt and Zarb proposed that implants can retain the bone by applying tensional forces, while the mucosal-borne prosthesis accelerates bone resorption due to compressive forces with a mean annual bone loss [36].

Based on two-dimensional radiographs, the included studies report alveolar bone development following MSI placement. Lateral bone levels can be evaluated with greater precision using advanced three-dimensional imaging modalities like computed tomography, compared to the limitations of traditional two-dimensional radiography. Moreover, understanding the formation of new bone around MSIs necessitates histological analysis of the targeted area. However, performing such studies in humans poses significant ethical challenges. Consequently, additional histologic investigations using animal models could provide valuable insights [37].

The aesthetic outcomes are also deemed satisfactory across multiple studies, addressing a key concern for young patients. Maintaining an aesthetically acceptable smile during critical psychosocial development stages enhances patient confidence and overall quality of life. Compared to traditional removable prostheses or fixed dental prostheses, MSI-supported pontics demonstrate superior functionality, with improved mastication, speech, and comfort reported in several cases [25]. Additionally, the non-invasive nature of MSIs, which avoids the enamel reduction required for fixed prosthetics, represents a significant advantage [15]. On the other hand, a limitation of fixed prostheses is the requirement for the presence of healthy and hard tissue in the adjacent elements to ensure optimal and long-lasting adhesion [38].

One notable advantage of MSI-supported pontics is their adaptability to the patient’s ongoing growth. In contrast, the risk of conventional dental implants is infraocclusion as the surrounding bone and dentition develop [39]. The use of dental implants in growing patients has been extensively studied, mainly focusing on conventional diameter implants. Studies indicate that infraocclusion is the primary issue, leading to challenging prosthetic management in adulthood [19]. Multiple studies have explored the potential of mini-implants in growing patients following dental avulsion. According to a 2019 review, there is insufficient evidence to advise against their use, with infraocclusion being the only documented adverse effect [40].

MSIs are removable and replaceable, making them ideal for temporary restoration. This adaptability allows clinicians to defer permanent solutions until craniofacial growth is complete, ensuring better long-term outcomes. Moreover, the flapless insertion techniques employed in some studies [23,32] reduce patient morbidity, post-operative discomfort, and recovery time. These attributes make MSI placement particularly suitable for younger patients, who may be less tolerant of invasive procedures. However, the lack of direct comparison with other transitional solutions in the reviewed studies limits a conclusive assessment of relative efficacy.

Despite these advantages, the studies also reveal notable complications. Prosthetic discoloration emerges as a recurring issue, highlighting the limitations of commonly used materials such as composite resin [11,13]. Long-term discoloration compromises aesthetic outcomes and may necessitate frequent replacement, increasing both cost and patient burden. Other complications are gingival impingement and minor diastema formation [13,32], potentially attributable to suboptimal pontic design or MSI positioning. While these issues were infrequent, they underscore the importance of meticulous treatment planning and execution. Additionally, the failure of one MSI due to mobility shortly after placement [23] raises questions about the factors influencing implant stability, including surgical protocols, MSI dimension, patient compliance, and individual anatomical variations.

A significant finding of this review is the heterogeneity in MSI dimensions, placement techniques, loading protocols, and retention strategies. MSI diameters ranged from 1.4 mm to 2.5 mm, with lengths typically between 8 mm and 10 mm when reported [16,31,32]. Similarly, retention methods varied widely, including resin-bonded prosthetics, temporary cement, and fixed retainers. This variability complicates efforts to draw generalizable conclusions and highlights the need for standardized guidelines [16]. It is crucial to develop a protocol designed to forecast the long-term success and survival of MSIs and supported pontics. Another critical limitation is the predominance of case reports and case series among the included studies. The absence of randomized controlled trials (RCTs) and control groups reduces the strength of the evidence and precludes definitive comparisons with alternative treatments. This gap represents a significant obstacle to establishing MSI-supported pontics as a routine clinical practice.

Despite these limitations, the findings suggest that MSI-supported pontics are a viable option for managing maxillary lateral incisor agenesis in growing patients [11,13,36]. Their ability to preserve alveolar bone, maintain root spaces, and support continuous bone remodeling during growth while simultaneously avoiding damage to adjacent teeth makes them particularly attractive for temporary use in growing patients. This advantage is especially relevant for ensuring optimal conditions for future definitive restorations [41]. However, clinicians should carefully consider patient-specific factors such as growth stage, oral hygiene, and aesthetic demands when selecting this approach. Given the potential for complications, regular follow-up and patient education to manage and mitigate potential complications effectively is essential to ensure long-term success. While pontic characteristics and the surgical techniques employed are important, maintaining routine oral hygiene remains a crucial factor for ensuring the long-term success and durability of implant therapy [42]. The primary tool suggested for home oral care was a standard toothbrush combined with triclosan-containing toothpaste, along with interproximal cleaning aids for all types of restorations. Additionally, irrigation devices were often recommended [43]. Patients and caregivers should be educated about the risks of discoloration and the possibility of prosthetic replacement. Additionally, proper planning of MSI placement and pontic design can minimize issues such as gingival impingement and diastema formation.

The limitations of this scoping review include the reliance on case reports and case series without randomized controlled trials (RCTs), the absence of control groups, the variability in clinical protocols across the selected studies, the limited duration of follow-up, the lack of consideration of patient-reported outcomes, such as comfort or long-term satisfaction, which could influence treatment choices. Furthermore, the main limitations of this study are its nature as a scoping review rather than a systematic review, which inherently restricts the depth of evidence synthesis and the lack of a detailed quality assessment of the included studies.

This review highlights several potential directions for future research. It remains a scoping review rather than a systematic one, as no quality assessment of the included studies was conducted. High-quality RCTs with larger sample sizes and longer follow-up periods are urgently needed to validate the efficacy of MSI-supported pontics and establish standardized protocols. Such studies should include control groups to enable direct comparisons with conventional treatments, such as removable prostheses and fixed dental prostheses. These treatments, although widely used, have significant limitations, including patient discomfort, speech, and soft tissue irritation. It is important to determine whether mini-implants complicate case resolution or provide similar or better outcomes to other temporary solutions like removable prostheses or Maryland bridges fixed dental prostheses [19].

These comparisons would allow clinicians to better assess the relative advantages of MSI-supported pontics in terms of functionality, patient comfort, and preservation of dental structures. Exploring novel prosthetic materials can improve durability and prevent discoloration, while advances in digital planning and 3D printing may enhance MSI precision and pontic fabrication, reducing complications. Additionally, assessing patient-reported outcomes would offer valuable insights into overall satisfaction and quality of life.

## 5. Conclusions

MSI-supported pontics offer significant advantages for the transitional management of maxillary lateral incisor agenesis, particularly in growing patients. They provide superior aesthetics and functionality, preserve alveolar bone, adapt to craniofacial growth, and avoid enamel reduction of adjacent teeth. Their removable nature ensures a flexible, minimally invasive solution. However, protocol variability and limited evidence underscore the need for further research. With advancements and standardization, MSI-supported pontics could become a key component of transitional dental care for young patients.

## Figures and Tables

**Table 1:** Search strategy for each database.

Database	Search Strategy
PubMed	((mini-implant OR mini-screw OR micro-screw OR transcortical mini-screw OR temporary anchorage device OR tad OR mini-implant anchorage OR anchorage screw OR self-drilling mini-screw OR skeletal anchorage device OR mini-screw implant OR orthodontic mini-screw implant OR orthodontic mini-implant OR orthodontic mini-screw) AND ((missing lateral incisor OR absence lateral incisor OR absent lateral incisor OR lateral incisor agenesis OR lateral incisor agenesia OR congenitally missing lateral incisor) OR (transitional restoration OR transitional replacement OR temporary restoration OR temporary replacement OR replacement missing lateral anterior teeth OR replacement missing lateral incisor OR temporary pontic OR temporary crown OR bonded pontic OR supported pontic OR semi-permanent replacement)))
WOS	TS = (mini implant OR mini-screw OR micro screw OR transcortical mini-screw OR temporary anchorage device OR tad OR mini-implant anchorage OR anchorage screw OR self-drilling mini-screw OR skeletal anchorage device OR mini-screw implant OR orthodontic mini-screw implant OR orthodontic mini implant OR orthodontic mini-screw) AND (TS = (missing lateral incisor OR absence lateral incisor OR absent lateral incisor OR lateral incisor agenesis OR lateral incisor agenesia OR congenitally missing lateral incisor) OR TS = (transitional restoration OR transitional replacement OR temporary restoration OR temporary replacement OR replacement missing lateral anterior teeth OR replacement missing lateral incisor OR temporary pontic OR temporary crown OR bonded pontic OR supported pontic OR semi-permanent replacement))
Scopus	(“mini-implant” OR “mini-screw” OR “micro screw” OR “transcortical mini-screw” OR “temporary anchorage device” OR “tad” OR “mini-implant anchorage” OR “anchorage screw” OR “self-drilling mini-screw” OR “skeletal anchorage device” OR “mini-screw implant” OR “orthodontic mini-screw implant” OR “orthodontic mini implant” OR “orthodontic mini-screw”) AND ((“missing lateral incisor” OR “absence lateral incisor” OR “absent lateral incisor” OR “lateral incisor agenesis” OR “lateral incisor agenesia” OR “congenitally missing lateral incisor”) OR (“transitional restoration” OR “transitional replacement” OR “temporary restoration” OR “temporary replacement” OR “replacement missing lateral anterior teeth” OR “replacement missing lateral incisor” OR “temporary pontic” OR “temporary crown” OR “bonded pontic” OR “supported pontic” OR “semi-permanent replacement”)))
Cochrane Library	(mini NEXT implant OR mini-screw OR micro NEXT screw OR transcortical NEXT mini-screw OR temporary NEXT anchorage NEXT device OR tad OR mini NEXT implant NEXT anchorage OR anchorage NEXT screw OR self NEXT drilling NEXT mini-screw OR skeletal NEXT anchorage NEXT device OR mini-screw NEXT implant OR orthodontic NEXT mini-screw NEXT implant OR orthodontic NEXT mini NEXT implant OR orthodontic NEXT mini-screw) in Title Abstract Keyword AND (missing NEXT lateral NEXT incisor OR absence NEXT lateral NEXT incisor OR absent NEXT lateral NEXT incisor OR lateral NEXT incisor NEXT agenesis OR lateral NEXT incisor NEXT agenesia OR congenitally NEXT missing NEXT lateral NEXT incisor) OR (transitional NEXT restoration OR transitional NEXT replacement OR temporary NEXT restoration OR temporary NEXT replacement OR replacement NEXT missing NEXT lateral NEXT anterior NEXT teeth OR replacement NEXT missing NEXT lateral NEXT incisor OR temporary NEXT pontic OR temporary NEXT crown OR bonded NEXT pontic OR supported NEXT pontic OR semi NEXT permanent NEXT replacement) in Title Abstract Keyword—(Word variations have been searched)

**Table 2:** Main characteristics of the studies.

Authors	Study Design	Patients (n)	Patients Age (Years)	Gender	Lateral Incisor’s Agenesis
Saha 2023 [32]	Case Report	1 Patient	12 Years	Female	Bilateral
Rathi 2023 [31]	Case Report	1 Patient	12 Years	Female	Unilateral
Ciarlantini and Melsen 2017 [13]	Case Series	5 Patients	13 Years 10 Years 11 Years 13 Years 11 Years	Female Female Male Male Female	Unilateral Bilateral Unilateral Unilateral Unilateral
Kalia 2015 [16]	Case Report	1 Patient	16 Years	Female	Unilateral
Cope and McFadden 2014 [11]	Case Series	2 Patients	11 Years 12 Years	Female Female	Unilateral Unilateral
Graham 2007 [23]	Case Series	2 Patients	14 Years 16 Years	Female Female	Unilateral Unilateral

**Table 3:** Mini-screw implant and prosthetic component characteristics of the included studies.

Authors	MSIs’ Number	MSI Diameter (mm)/ Length (mm)	MSI Positioning/ Insertion Protocol	MSI Loading	Prosthetic Retention	Type of Prosthetic Component	Type of Retention
Saha 2023 [32]	2	1.5/10	Perpendicular to the alveolar crest; parallel to the central incisors’ long axis/flapless technique	After 4 weeks	NS	Composite resin crown	Hawuley restraint plate
Rathi 2023 [31]	1	2.5/10	Perpendicular to the alveolar crest/full-thickness flap	NS	Temporary cement (zinc phosphate cement)	Composite resin crown	NS
Ciarlantini and Melsen 2017 [13]	6	NS/NS	Palatally perpendicular to the alveolar process/NS	Immediately after MSI placement	Resin bonded on a 0.021 × 0.025 stainless steel wire section from the head of the MSI	Composite resin crown	The canine and the central incisor were splinted with the adjacent teeth but not with the pontic
Kalia 2015 [16]	1	1.4/10	Perpendicular to the alveolar crest/NS	NS	Permanent cement (glass ionomer cement)	Porcelain fused to metal crown	Maxillary fixed retainer that did not include the MSI crown
Cope and McFadden 2014 [11]	2	2.2/NS	Perpendicular to the alveolar crest/full-thickness flap	Immediately after MSI placement	Temporary cement	Provisional polycarbonate crown and ceramic crown	NS
Graham 2007 [23]	3	2/8 and 10; 2/10	Perpendicular to the alveolar crest/flapless technique; Perpendicular to the alveolar crest/flapless technique	Immediately after MSI placement; Immediately after MSI placement	Resin bonded; Resin bonded	Resin crown (denture tooth); Resin crown (denture tooth)	Clear plastic retainer and a lingual fixed retainer (including the pontic); Clear plastic retainer and a lingual fixed retainer (including the pontic)

NS: not specified.

**Table 4:** Outcomes during the follow-up time.

Authors	Follow-Up Time	Clinical Outcomes	Complications
Saha 2023 [32]	9 Months	Positive: no implant mobility; no soft tissue inflammation	1 mm diastema between 1.1 and 2.1
Rathi 2023 [31]	24 Months	Positive: no marginal bone loss; no bleeding on probing	NS
Ciarlantini and Melsen 2017 [13]	5 Years	Positive: bone density maintained; vertical development of the alveolar process	Prosthetic discoloration in 1 patient; gingival impingement in 2 patients
Kalia 2015 [16]	1 Year	Positive: no alveolar bone loss around MSI; favorable esthetic	NS
Cope and McFadden 2014 [11]	8 Years 27 Months	Positive: no infraocclusion; no bone defect Positive: no infraocclusion; coronally bone growth	Provisional crown discoloration
Graham 2007 [23]	1 Months and 14 Months 8 Months	Negative: mini-screw mobility and positive: no pain and mini-screw stability Positive: no pain and mini-screw stability	Failure; NS NS

NS: not specified.

## Data Availability

The data presented in this study are available in the article.
